# Nitrogen Deposition Effects on Diatom Communities in Lakes from Three National Parks in Washington State

**DOI:** 10.1007/s11270-013-1857-x

**Published:** 2014-02-01

**Authors:** Richard W. Sheibley, Mihaela Enache, Peter W. Swarzenski, Patrick W. Moran, James R. Foreman

**Affiliations:** 1 US Geological Survey, Tacoma, WA 98402 USA; 2 Academy of Natural Sciences, Philadelphia at Drexel University, Philadelphia, PA 19103 USA; 3 Present Address: NJ Department of Environmental Protection, Trenton, NJ 08625 USA; 4 US Geological Survey, Santa Cruz, CA USA

**Keywords:** Critical loads, N deposition, Diatoms, DIN/TP, National Parks, Lake chemistry

## Abstract

The goal of this study was to document if lakes in National Parks in Washington have exceeded critical levels of nitrogen (N) deposition, as observed in other Western States. We measured atmospheric N deposition, lake water quality, and sediment diatoms at our study lakes. Water chemistry showed that our study lakes were ultra-oligotrophic with ammonia and nitrate concentrations often at or below detection limits with low specific conductance (<100 μS/cm), and acid neutralizing capacities (<400 μeq/L). Rates of summer bulk inorganic N deposition at all our sites ranged from 0.6 to 2.4 kg N ha^−1^ year^−1^ and were variable both within and across the parks. Diatom assemblages in a single sediment core from Hoh Lake (Olympic National Park) displayed a shift to increased relative abundances of *Asterionella formosa* and *Fragilaria tenera* beginning in the 1969–1975 timeframe, whereas these species were not found at the remaining (nine) sites. These diatom species are known to be indicative of N enrichment and were used to determine an empirical critical load of N deposition, or threshold level, where changes in diatom communities were observed at Hoh Lake. However, N deposition at the remaining nine lakes does not seem to exceed a critical load at this time. At Milk Lake, also in Olympic National Park, there was some evidence that climate change might be altering diatom communities, but more research is needed to confirm this. We used modeled precipitation for Hoh Lake and annual inorganic N concentrations from a nearby National Atmospheric Deposition Program station, to calculate elevation-corrected N deposition for 1980–2009 at Hoh Lake. An exponential fit to this data was hindcasted to the 1969–1975 time period, and we estimate a critical load of 1.0 to 1.2 kg N ha^−1^ year^−1^ for wet deposition for this lake.

## Introduction

Human alteration of global biogeochemical cycles has been well documented and observed changes have been dramatic over the last century (Galloway et al. 1995; Schlesinger 1997; Vitousek et al. 1997). In particular, nitrogen (N) cycles have been extensively modified through increases in fossil fuel combustion from vehicles and industry, fertilizer production, and application to the land surface and livestock operations, with the largest changes occurring within the last 50–60 years (Galloway et al. 1995; Vitousek et al. 1997; Tilman et al. 2001). The amount of fixed N (the conversion of N_2_ gas to ammonia or other reduced forms), which is conveyed atmospherically to terrestrial and aquatic ecosystems, has doubled worldwide (Vitousek et al. 1997; Galloway et al. 2008; Swackhamer et al. 2004). In fact, a significant fraction of “new” N to many ecosystems within the USA comes from direct atmospheric deposition of N (Swackhamer et al. 2004; Greaver et al. 2012). Atmospheric deposition of N is of particular concern because although effects are usually greatest near the sources of pollution (Fenn et al. 2003a), impacts of N enrichment from deposition can be seen in areas located far from pollution sources (Saros et al. 2003; Elser et al. 2009; Greaver et al. 2012) making increased N deposition a local, regional, and national issue. Even remote ecosystems of National Parks have not been spared from N-associated degradation (Wolfe et al. 2001; Baron 2006; Saros et al. 2003, 2011). As a result, there has been a significant increase in studies pertaining to the effects and impacts of increased N deposition on terrestrial and aquatic ecosystems in the USA in the last 15 years (Fenn et al. 2003b; Sickman et al. 2003; Elser et al. 2009; Pardo et al. 2011; Greaver et al. 2012).

Increases in atmospheric N deposition can have many negative effects on both terrestrial and aquatic ecosystems. In terrestrial systems, effects include higher foliar and soil N content, higher rates of soil mineralization and nitrification, changes in plant communities leading to lowering of biodiversity, and increases in soil greenhouse gas emissions (Fenn et al. 2003b; Baron et al. 2000; Greaver et al. 2012). In aquatic ecosystems, deleterious impacts include increases in nitrate (NO_3_^−^) runoff, and lake and stream NO_3_^−^ concentrations, which may induce increases in algal productivity, lowering of dissolved oxygen concentrations, lake and stream acidification, reduced water clarity, and shifts in N and P limitation of lake phytoplankton (Baron et al. 2000, 2011; Fenn et al. 2003b; Sickman et al. 2003; Elser et al. 2009). Besides these direct effects on terrestrial and aquatic systems, indirect effects from increased N deposition also exist. For example, as N deposition increases in terrestrial systems, so can forest productivity, resulting in larger amounts of organic matter standing stocks on forest floors, potentially increasing forest fire risk (Greaver et al. 2012). In aquatic systems, increases in algal productivity can enhance bioaccumulation of persistent organic pollutants as algae contain a high lipid content (Swackhamer et al. 2004) and efficient grazing by zooplankton can increase the possibility that organic pollutants are passed onto higher trophic levels in the aquatic food web (Swackhamer and Skoglund 1993; Skoglund and Swackhamer 1994).

Although the effect of increased N deposition is widespread across many ecosystem types, high elevation lakes are considered particularly sensitive to these increases. High elevation lakes are typically oligotrophic, so even small changes in N inputs can induce substantial changes in productivity and N cycling (Sickman et al. 2003). In addition, high-elevation lakes tend to receive more precipitation, have poorly developed soils, are more sparsely covered with vegetation, and have higher proportions of exposed bedrock compared to more forested locations (Fenn et al. 1998; Larson et al. 1999; Baron et al. 2005). All of these factors lead to more efficient delivery of N from precipitation to the surface water of these lakes. As many high elevation lakes in National Parks are oligotrophic, and have basin characteristics similar to those outlined above (Clow et al. 2003; Nanus et al. 2009), federal land managers are concerned about how current N deposition is impacting resources within their jurisdictions.

One method of establishing the impact of N deposition on ecosystems is by estimating an empirical critical load. A “critical load” is defined as “the deposition of a pollutant below which no detrimental ecological effect occurs over the long term based on present knowledge” (Pardo et al. 2011). This level can be used by land managers to establish a benchmark for protecting their natural resources (Porter et al. 2005; Burns et al. 2008). Different critical load values can be defined for the same system for different specific effect endpoints. For example, the critical load for acidification and eutrophication of the same lake could occur at different levels of N deposition. The critical load approach has been widely used in Europe for documenting the extent of freshwater acidification (Posch et al. 1995, 2001) and has been recently gaining interest in the United States (Burns et al. 2008; Baron et al. 2011; Pardo et al. 2011). In this paper, we focus on determining a critical load for N deposition in high elevation surface waters in Washington State using lake sediment diatoms. Diatoms are unicellular aquatic microscopic algae that respond rapidly to changes in water chemistry mediated by environmental change. As they have siliceous cell walls that are taxonomically identifiable to species level, and are preserved well in lake bottom sediments, diatoms produce an archive of environmental history allowing for past ecological inferences, including N enrichment (Wolfe et al. 2001, 2003; Saros et al. 2003, 2011; Battarbee et al. 2012). The analysis of historical changes in diatom community structure is a powerful tool and can provide insight into if and when a change from oligotrophic to more nitrophilic species has occurred.

Critical loads of N deposition for lake sediment diatoms have been established in several western states. Research at Rocky Mountain National Park in Colorado identified a critical load for wet N deposition of 1.5 kg N ha^−1^ year^−1^ for high alpine lakes (Baron 2006). Similarly, Saros et al. (2011) identified a critical load for diatoms at 1.4 kg N ha^−1^ year^−1^ at sites in Yellowstone National Park (Wyoming) and the eastern Sierra Nevada mountains (California). There are six long-term National Atmospheric Deposition Program (NADP) sites in Washington State and data from these sites show wet deposition values can be at or above the 1.5 kg N ha^−1^ year^−1^ critical load from these studies (http://nadp.sws.uiuc.edu/sites/sitemap.asp?state=wa). As a result, identifying a critical load for Washington State National Parks was identified as a high research priority for the region (Waddell and Greenwood 2006). For this study, we sampled water quality and analyzed diatom assemblages in sediment cores from several high elevation lakes in three national parks in Washington State. Data from this effort were used to (1) examine changes in sediment diatom communities related to N deposition, (2) identify the time period in which this change occurred, and (3) use elevation-corrected NADP data to determine the level of N deposition that induced these changes in diatom communities. In addition, we estimated N deposition at our sites using field-deployed bulk deposition collectors during summer to provide a snapshot of bulk N deposition during the study.

## Study Sites

**Fig. 1:**
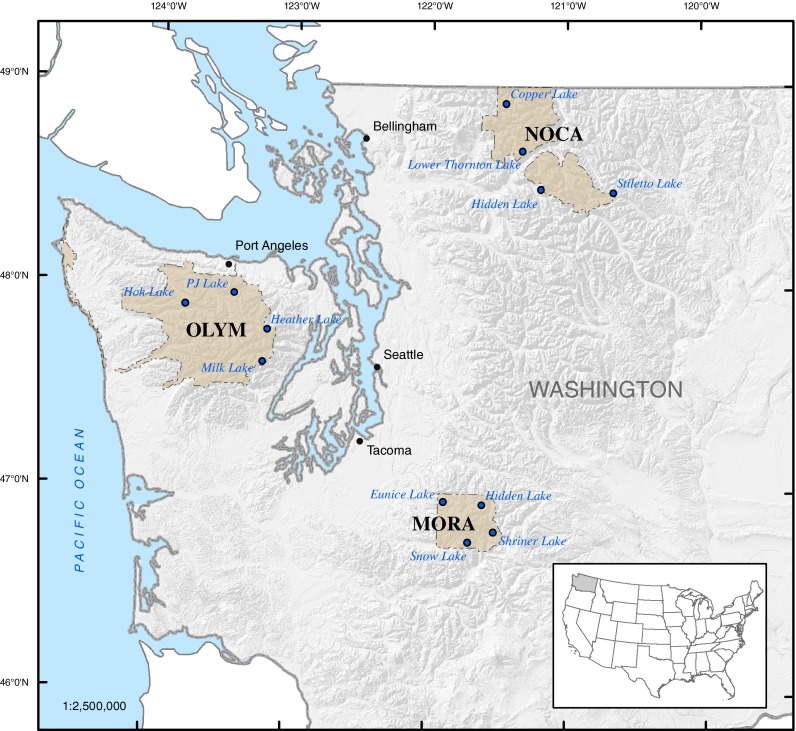
Location of study sites in Washington State (*MORA* Mt. Rainier, *NOCA* North Cascades, *OLYM* Olympic); individual study lakes given by *black circles*

A total of 12 lakes were selected for this study from Mt. Rainier (MORA), North Cascades (NOCA), and Olympic (OLYM) National Parks in Washington State (Fig. 1). Sites were selected to maximize observation of potential impacts from N deposition. We targeted lakes above the tree line (above ∼1,200 m) at each park to reduce the data variability associated with N fixation and geochemical processing by subalpine plant species, namely the red alder (*Alnus* sp.) (Rojas et al. 2001). Lakes with significant fish populations were avoided to reduce bioturbation of the sediment and to minimize changes in lake N cycling caused by the presence of fish in order to better isolate N deposition as a cause for diatom changes. We focused on lakes that were less than 25 ha with a maximum depth of 10 m or greater to balance the logistics of water sampling, sediment sampling and reduce disturbances from wildlife while still targeting “typical” lake characteristics within each park. As the main goal of this study was to assess impacts from atmospheric N deposition, lakes that currently and historically were low in nutrients (oligotrophic) were preferred. A summary of site characteristics for each lake is provided in Table 1.

**Table 1:** Physical properties of study lakes

Site name	USGS site ID^a^	Latitude^b^	Longitude	Elevation (m)	Area (ha)	Max. depth (m)
Mount Rainier National Park (MORA)						
Eunice	12091956	46.95563	121.87722	1,634	5.32	20
Hidden MORA	12096700	46.94181	121.59812	1,807	2.12	7
Shriner	14223825	46.80900	121.51453	1,490	1.67	4
Snow	14224590	46.75759	121.69825	1,426	2.39	10
North Cascades National Park (NOCA)						
Copper	12215650	48.91818	121.45149	1,604	5.22	20
Hidden NOCA	12181450	48.49583	121.18885	1,747	24.98	79
Lower Thornton	12178700	48.68420	121.32806	1,367	22.30	33
Stiletto	12450880	48.48197	120.65632	2,071	4.01	26
Olympic National Park						
Heather	12047660	47.78508	123.17788	1,589	0.4	7
Hoh	12040680	47.89872	123.78588	1,384	7.44	15
Milk	12053810	47.62525	123.20604	1,435	1.1	14
PJ	12047150	47.96069	123.42837	1,384	0.8	6

^a^USGS site number is unique to this site and allows for access to data through NWIS, the national water information system at http://waterdata.usgs.gov/nwis

^b^Latitude and longitude of site, in decimal degrees, referenced to NAD 27

## Field and Analytical Methods

### Bulk Atmospheric Deposition Estimates

Bulk atmospheric deposition of N and S (sulfur) were estimated at each of the 12 study lakes using a low-maintenance, passive deposition collector developed by Fenn et al. (2002). This simple collector consists of a funnel attached to a small-diameter column that contains an ion exchange resin (IER); as rain water passes through the IER column, anions and cations are sorbed onto the resin. This method estimates the bulk (or total wet plus dry) deposition during the deployment period as the funnels are placed out in the open away from the influence of vegetation. This method of collection does not completely incorporate deposition from fog, other than what might be captured on the inside of the funnel and drained through the column. The IER columns were deployed during summer and collected in early autumn in 2008 when the columns were retrieved and extracted for analysis of sorbed ions in the laboratory. A set of five IER columns were spread out around each lake basin to account for site variability. Additionally, a control column that consisted of an IER column capped at both ends was left in the field at each lake basin site during the same period as the active columns. The control column from each site was used to account for the potential release of ammonia ions from the IER from natural diurnal heating and cooling as described by Fenn et al. (2002).

In the laboratory, each column was extracted using a 2 N KI solution, and the extract was analyzed for NO_3_^−^, ammonia (NH_4_^+^), orthophosphate (PO_4_^−3^), and sulfate (SO_4_^−2^); however, phosphate was only used to identify potential contamination from birds and deposition of phosphorus was not quantified for this study. Ammonia was analyzed using a continuous flow analyzer, and NO_3_^−^, SO_4_^−2^, as well as PO_4_^−3^ were analyzed using ion chromatography. Details of the laboratory analysis methods and results of extensive field tests using this method are available in Fenn et al. (2002, 2009) and Simkin et al. (2004). Quality control samples used to verify the usability of the IER data included field and laboratory blank columns (for background correction), and IER columns spiked with low and high levels of N and S. A detailed analysis of all quality control data for estimating atmospheric deposition in this study can be found in Sheibley et al. (2012).

The mass loading from the set of five columns at each site were averaged and converted to a mass per time per area (kilograms per hectare per year) using the area of the funnel and the total number of days the columns were deployed in the field. All deposition rates determined from IER columns are considered “summer rates” that are scaled to a common time period (a year in this case) in order to facilitate comparison across sites. These rates do not include deposition during other seasons and therefore do not reflect the total annual deposition rate.

### Lake Water Quality

Samples of surface water were collected at each of the study lakes to provide a snapshot of lake water quality. Samples were collected in the summer months after spring runoff, and before fall turnover and represent “base flow” for comparisons across sites. These samples were collected prior to sediment coring (described below), from the water surface (upper 1 m of depth), and above the deepest part of the lake. Analytes determined include the following: nutrients (NO_2_+NO_3_^−^, NH_4_^+^, and PO_4_^−3^), total N (TN), and total phosphorus (TP), chlorophyll-*a* (Chl-*a*), and acid neutralizing capacity (ANC). Samples collected for TN and TP were unfiltered and represent total dissolved and particulate N and P, respectively. Samples for Chl-*a* were also unfiltered, while samples for NO_2_+NO_3_^−^, NH_4_^+^, and PO_4_^−3^ were filtered in the field using a 60-cm^3^ syringe and a 0.45- or 0.22-μm membrane filter. Samples were kept cool during transport from each field site and nutrient samples were frozen immediately upon return to laboratory. The ANC of each lake was determined in the laboratory by titration and calculated using the inflection point method (Rounds 2006). Samples collected for Chl-*a* were filtered onto a 0.45-μm glass-fiber filter disk, folded, wrapped in aluminum foil, and frozen until analysis. Nutrient data were used to calculate the mass ratios of DIN (NH_4_^+^+NO_3_^−^) to TP (DIN/TP) of the surface water at the time of core collection. This ratio has been shown to be a good indicator of N or P limitation of lakes as it provides a useful measure of the nutrient supply available to phytoplankton (Bergstrom 2010; Morris and Lewis 1988; Sickman et al. 2003).

Chl-*a* was analyzed by the US Geological Survey (USGS) National Water Quality Laboratory in Lakewood, Colorado, using established methods (EPA method 445.0; Arar and Collins 1997). Nutrient samples were analyzed by the Cooperative Chemical Analytical Laboratory at Oregon State University, Corvallis, using a continuous-flow autoanalyzer following standard methods (Ameel et al. 1993; American Public Health Association 2005). All water quality data for this study were censored at either the laboratory reporting limit (Childress et al. 1999) for Chl-*a*, or at the method detection limit for nutrients (Cooperative Chemical Analytical Laboratory 2012).

### Sediment Coring

Lake sediment cores were collected using a gravity corer (UWITEC, Inc.) and a pontoon boat and were collected from the deepest part of each lake. Cores of sufficient length (target 30 cm) with a clear and level sediment-water interface were stored upright on the boat until a total of four cores were collected. Two cores, based on visual similarities and overall length, were used for diatom analysis and dating. Cores were sectioned in 0.5-cm increments for the first 10 cm, and then every 1 cm thereafter until expended. Sediment slices were collected using a calibrated, screw-type, stainless steel extruder using the same equipment and field procedures used by Landers et al. (2008) and collected into clean, zip-top plastic bags. Sectioned samples were kept in the dark and refrigerated to prevent further chemical or metabolic processing.

### Diatom Analysis

After sectioning the lake core, a subsample from each zip-top plastic bag was removed for diatom analysis. Diatom subsamples were obtained by gently stirring the sediment sample with a metal spatula, and scooping out a small amount of wet sediment for placement in a small scintillation vial. A total of 1–3 mL of wet material was used for diatom sample preparation.

Diatom samples were refrigerated until they were shipped to the phycology laboratory at the Patrick Center for Environmental Research at the Academy of Natural Sciences of Drexel University, Philadelphia, Pennsylvania (ANSP). Sediment samples were digested with nitric acid in a microwave apparatus and mounted with Naphrax™ on glass slides. All samples were processed and analyzed following the ANSP Phycology Section standard operating procedures from Charles et al. (2002). Two permanent diatom slides were made from each sediment sample. A suspension of cleaned diatom frustules was diluted repeatedly to remove residual acid and a subsample was spread evenly onto glass coverslips and allowed to dry. The glass coverslips were then mounted on glass slides using Naphrax™ mounting medium.

A minimum of 500 diatom valves were identified for each sample at a magnification of at least 1,000 times using a Leica™ DM LB2 microscope equipped with differential interference contrast. The diatom taxonomy primarily was based on the references of Krammer and Lange-Bertalot (1986; 1988, 1991a, b), Cumming et al. (1995), Camburn and Charles (2000), Tanaka (2007), and taxonomic harmonization within the USGS National Water Quality Assessment Program reports (Academy of Natural Sciences 2012). Each diatom species identified was assigned a unique North American Diatom Ecological Database identifier in order to track and to adjust for any future changes in diatom taxonomy that might occur.

Diatom samples from the top and bottom slice from all ten lakes were examined for taxon relative abundance and known indicators of N enrichment namely, *Asterionella formosa* and *Fragilaria crotonensis* (Wolfe et al. 2001; Saros et al. 2005, 2011). From this information, combined with N deposition estimates, four lakes were selected for a more detailed down-core analysis of diatom assemblages and corresponding sediment dating. This paper primarily presents diatom data from the four cores where detailed historical diatom communities were determined. Complete diatom assemblages from all sediment samples analyzed for this study are available elsewhere (Sheibley et al. 2012).

### Sediment Geochronologies

Sediment dates were determined using natural and artificial (man-made, derived from past nuclear weapons testing) radionuclides following established methods (Ivanovich and Harmon 1992; Swarzenski et al. 2003; Swarzenski 2007). Of these radionuclides, lead-210 (^210^Pb) and cesium-137 (^137^Cs) have been used extensively to reconstruct records of sedimentation for relatively young (less than 100 years) sediment deposits (Appleby and Oldfield 1978; Appleby 2008). The ^210^Pb removed from the water column by particles will have a higher ^210^Pb activity than the activity of its indirect radiogenic parent, radium-226 (^226^Ra). As a result, there are two pools of ^210^Pb—one is the parent-supported pool whose activity is the considered close to that of ^226^Ra, and the other is the unsupported (termed “excess”) ^210^Pb pool, or “xs” ^210^Pb. Sediment geochronologies were developed for each lake from the excess ^210^Pb profiles.

Sediment cores for four of the ten lakes were dated using the constant rate of supply model (Appleby and Oldfield 1978; Appleby 2008). After taking the diatom subsample, the remaining sediment was dried at 60 °C for several days and ground to a fine powder using a mortar and pestle. Bulk densities of each sediment slice were determined from changes in wet and dry weights and the known volume of the sediment slice. A known amount of the powdered samples were sealed in vials and activities of ^210^Pb, ^137^Cs, and ^226^Ra were determined using a high purity germanium (HPGe) well detector (Princeton Gamma Tech Instruments, Inc.) using established methods (Swarzenski et al. 2006; Swarzenski et al. 2008). Efficiency curves for the HPGe well detectors were obtained using a suite of International Atomic Energy Agency (IAEA) gamma ray spectrometry reference materials (RGU-1, RGTh-1, and IAEA-300) using the same volume of standard as the sediment samples.

### Estimation of Critical Load

Using the ^210^Pb and ^137^Cs data, we identified the time period where changes in diatom indicators (*A. formosa* and *F. crotonensis*) first doubled in relative abundance from background levels and were at least 5 % of the total abundance (sensu Saros et al. 2011). We used NADP data near any impacted site to hindcast what deposition was when these diatom shifts took place to identify our critical load using a combination of several previous methods (Baron 2006; Baron et al. 2011; Saros et al. 2011). First, as all the NADP data in Washington is at low elevation, we needed to correct the deposition data to the elevation of any affected lake. Elevation-corrected deposition data was generated by taking Parameter-Elevation Regressions on Independent Slopes Model (PRISM) precipitation data at the target lake and then multiplied by the annual precipitation concentration data for N from the closest NADP station. PRISM provides continuous high-resolution GIS layers of historic and contemporary precipitation for the US that can take into account orographic precipitation effects common to mountain terrains found in Washington State (www.prism.oregonstate.edu). NADP data in Washington was not collected prior to 1980, so for time periods prior to this date, we fit an exponential equation to our elevation-corrected deposition data and hindcasted to the time period defined by the diatom record (sensu Baron 2006; Saros et al. 2011). We then solved this equation for each year of our defined time period for diatom change and took the average and standard deviation to define our critical load. We only used NADP data that passed the program’s four quality control criteria (http://nadp.sws.uiuc.edu/documentation/completeness.asp) which resulted in 8 years during the 1980–2009 time period for which we did not use the reported NADP wet deposition value. Both Baron (2006) and Saros et al. (2011) included a preindustrial “anchor point” of 0.5 kg N ha^−1^ year^−1^ for the Western USA based on work of Holland et al. (1999) when hindcasting deposition in their studies. Here, we provide a range for our critical load estimates by fitting our elevation-corrected NADP deposition data both with and without the same anchor point (0.5 kg N ha^−1^ year^−1^ in 1900) in order to provide a range in our estimate of the critical load.

### Statistical Analyses

We used an analysis of variance (ANOVA) to test for differences in deposition among lakes within each park (*n* = 4), as well as differences in average deposition across each park (*n* = 3) in order to evaluate differences in deposition within and between the three National Parks (S plus; TIBCO Software Inc., ver 8.1). Where a significant difference was detected, we used the Tukey test to identify which lake or park was causing these differences. All statistical tests used a 95 % confidence limit for significance. To examine how different or similar diatom communities were within and across parks, we evaluated whole diatom communities using multivariate routines from the Primer 6 statistical package (Clarke and Warwick 2001). Specifically, cluster analysis of relative abundance of diatom valve counts from surface and bottom core slices were evaluated by group average following standardization by total sample count, square root normalization and Bray–Curtis resemblance (McCune and Grace 2002).

In addition, detrended correspondence analysis (DCA) was performed on each core to summarize main gradients in diatom assemblages along the stratigraphic profile. DCAs were performed by segments with down-weighting of rare taxa and nonlinear scaling and included all taxa. DCA axis 1 scores were used to summarize the dominant gradient of diatom composition turnover (Hill and Gauch 1980). DCAs were performed using the “vegan” package of the program R (http://cran.r-project.org/web/packages/vegan/).

## Results

### Bulk Deposition and Water Chemistry

In general, park-averaged N and S summer bulk deposition was greatest at NOCA, with OLYM having the lowest values, and MORA lying in between (Table 2). Summer bulk inorganic N (sum of NO_3_^−^ and NH_4_^+^) deposition was variable across the four individual lakes in MORA ranging from 1.07 to 1.97 kg N ha^−1^ year^−1^. Summer bulk deposition for sulfate varied (1.05 to 1.90 kg S ha^−1^ year^−1^) across all sites as well. Although bulk deposition rates varied among lakes, there were no statistically significant differences across individual sites in MORA (ANOVA; *p* > 0.05). At NOCA, Stiletto Lake had consistently lower summer bulk deposition of N and sulfur compared to the other three lakes (*p* < 0.05). Stiletto Lake was the most eastern site at NOCA and the lower deposition was likely because this was a ‘dry’ site with about half the amount of precipitation compared to other NOCA sites (data not shown). Summer bulk inorganic N deposition ranged from 1.19 to 2.42 kg N ha^−1^ year^−1^, and summer bulk SO_4_^−2^ deposition ranged from 1.12 to 2.28 kg S ha^−1^ year^−1^ across all sites in NOCA. At OLYM, summer bulk inorganic N deposition ranged from 0.59 to 1.04 kg N ha^−1^ year^−1^ and summer bulk SO_4_^−2^ deposition ranged from 0.86 to 2.33 kg S ha^−1^ year^−1^. In general, NO_3_^−^ and NH_4_^+^ each contributed about half of the total summer bulk inorganic N deposition across all sites and all three parks. Results of orthophosphate analyses showed no indication of contamination of samples from bird waste (data not shown). Results from an ANOVA showed that park-averaged summer bulk NH_4_^+^ deposition at OLYM was significantly less than both NOCA and MORA (*p* < 0.05, *n* = 3) while both NO_3_^−^ and inorganic N summer bulk deposition were significantly different within each park (*p* < 0.05, *n* = 3), and SO_4_^−2^ summer bulk deposition was statistically similar across each park (*p* > 0.05, *n* = 3).

**Table 2:** Summary of bulk deposition data collected during Summer 2008

Site	Days deployed	NH_4_–N (kg ha^−1^ year^−1^)	NO_3_–N (kg ha^−1^ year^−1^)	Inorganic N^b^ (kg ha^−1^ year^−1^)	SO_4_–S (kg ha^−1^ year^−1^)
Eunice	85	1.07 ± 0.10	0.91 ± 0.12	1.97 ± 0.19	1.90 ± 0.44
Hidden MORA	76	0.58 ± 0.17	0.49 ± 0.18	1.07 ± 0.34	1.18 ± 0.38
Shriner^a^	86	0.95	0.56	1.51	1.05
Snow	71	0.51 ± 0.14	0.58 ± 0.12	1.09 ± 0.22	1.45 ± 0.44
Mt. Rainier NP average		0.73 ± 0.28	0.65 ± 0.22	1.38 ± 0.48	1.48 ± 0.49
Copper	66	1.06 ± 0.21	1.20 ± 0.11	2.27 ± 0.32	2.28 ± 0.40
Hidden NOCA	59	1.01 ± 0.07	1.02 ± 0.22	2.02 ± 0.19	1.71 ± 0.58
Lower Thornton	72	1.10 ± 0.21	1.32 ± 0.19	2.42 ± 0.27	2.10 ± 0.70
Stiletto	71	0.46 ± 0.41	0.73 ± 0.24	1.19 ± 0.40	1.12 ± 0.27
North Cascades NP average		0.93 ± 0.34	1.09 ± 0.29	2.02 ± 0.55	1.84 ± 0.65
Heather	65	0.25 ± 0.15	0.45 ± 0.11	0.70 ± 0.10	0.86 ± 0.49
Hoh	68	0.49 ± 0.25	0.48 ± 0.15	0.97 ± 0.30	2.33 ± 0.69
Milk	80	0.49 ± 0.07	0.55 ± 0.12	1.04 ± 0.13	1.31 ± 0.32
PJ	60	0.25 ± 0.11	0.34 ± 0.04	0.59 ± 0.07	1.25 ± 0.17
Olympic NP average		0.37 ± 0.19	0.46 ± 0.13	0.83 ± 0.42	1.44 ± 0.70

^a^Only one resin column was retrieved; no SD calculated

^b^NH_4_+NO_3_

Surface water chemistry indicated that all our lakes were dilute, oligotrophic systems (Table 3). Specific conductance at MORA and NOCA lakes was consistently less than 10 μS/cm and all had ANC below 100 μeq/L, whereas OLYM lakes had higher ANC (284-374 μeq/L) and specific conductance (37-71 μS/cm). Nutrient concentrations in all parks were very low and often below the laboratory detection limits. Ammonia and PO_4_^−3^ were either at or below the detection limits for all samples except for PO_4_^−3^ concentration at Stiletto Lake. Nitrate was detected in most lakes, but it was still low ranging from below the detection limit to 7 μg N/L. Total N varied within and across parks ranging from 40 to 250 μg N/L and TP was never greater than 10 μg P/L. Productivity at these lakes was also low with Chl-*a* concentrations less than 1.0 μg/L at eight of the ten lakes where it was measured.

**Table 3:** Lake surface water quality data from study lakes

Site name	Sample date	Specific conductance (μS/cm)	pH	ANC (μeq/L)	NH_4_^+^ (mg N/L)	NO_3_^-^ (mg N/L)	Ortho-phosphate (mg P/L)	Total nitrogen (mg N/L)	Total phosphorous (mg P/L)	DIN:TP	Chl-a (μg/L)
Eunice	11/04/09	7.5	6.5	30	<0.01	0.002	<0.001	0.250	0.012	0.7	0.4E
Hidden MORA	08/19/10	15.8	6.7	46	<0.01	0.001	0.001	0.060	0.004	1.5	0.7E
Shriner	–^a^	–^a^	–^a^	–^a^	–^a^	–^a^	–^a^	–^a^	–^a^	–^a^	–^a^
Snow	10/07/09	12.9	5.4	10	<0.01	<0.001	<0.001	0.090	0.006	0.9	1.51E
Copper	09/22/09	5.6	6.1	26	<0.01	<0.001	<0.001	0.070	0.004	1.4	0.54
Hidden NOCA	09/24/09	5.6	6.7	30	<0.01	0.001	<0.001	0.050	0.002	2.8	0.23
Lower Thornton	09/23/09	10.3	6.9	50	<0.01	0.004	<0.001	0.100	0.003	3.0	0.8
Stiletto	08/26/09	9.4	6.3	90	<0.01	0.006	0.003	0.270	0.007	1.6	1.03
Heather	09/10/09	71.0	7.2	374	<0.01	0.007	0.001	0.060	0.005	2.4	0.62
Hoh	09/08/09	62.2	7.5	394	<0.01	0.001	<0.001	0.160	0.007	0.9	0.63
Milk	09/11/09	37.2	7.2	284	<0.01	<0.001	<0.001	0.040	0.004	1.4	0.28
PJ	–^a^	–^a^	–^a^	–^a^	–^a^	–^a^	–^a^	–^a^	–^a^	–^a^	–^a^

DIN = NH_4_ + NO_3_

*E* estimated, detected in sample, but below method detection limit

^a^No sample

Using half of the detection limit for censored data in Table 3, DIN/TP mass ratios were 1.5 or less (indicating N-limitation; Bergstrom 2010) at all the lakes in MORA, at Hoh and Milk lakes OLYM and at Copper Lake in NOCA; whereas Hidden, Lower Thornton, and Stiletto at NOCA and Heather Lake at OLYM indicated possible co-limitation of N and P (Table 3). None of the study lakes indicated strict P limitation (DIN/TP, >3.4; Bergstrom 2010) at the time of sampling.

### Sediment Dating and Diatom Analyses

A total of 56 sediment samples were analyzed for diatoms in top-bottom and stratigraphic sediment cores, with over 250 diatom species identified. Diatom remains were well preserved in all cores, including delicate valves of *Achnathaceae*; diatom valves did not display evidence of partial dissolution, thus we are confident that the observed species composition changes were true and not an artifact of dissolution. At each of the ten study lakes a set of top and bottom core samples were examined to look for whole community similarities as well as taxa known to be indicators of N enrichment *A. formosa* and *F. crotonensis* (Wolfe et al. 2001; Saros et al. 2005, 2011). This initial diatom analysis was used as a screening tool to identify four lakes for a more detailed down core analysis of historical diatom assemblages and corresponding sediment dating.

**Fig. 2:**
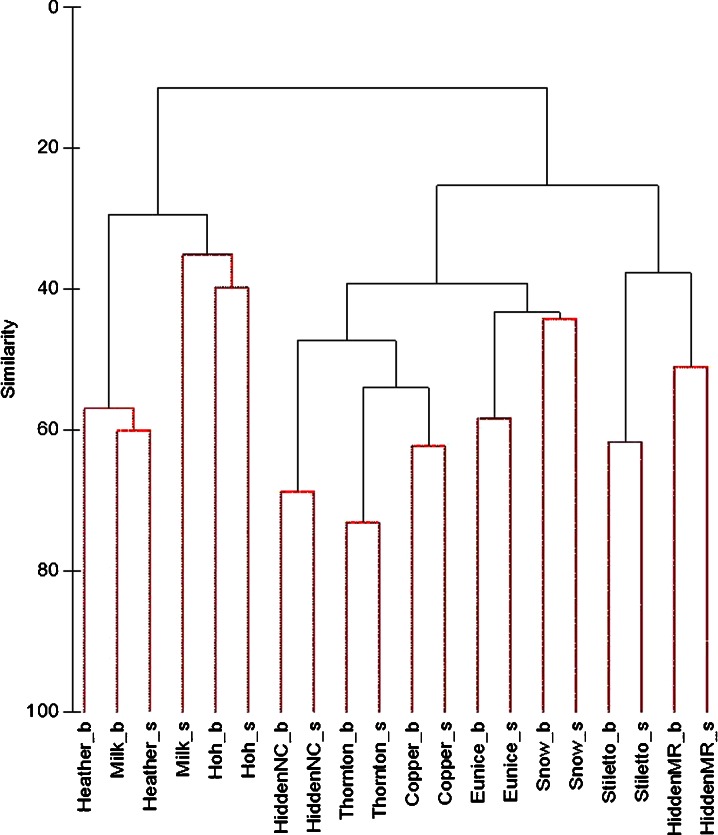
Similarity cluster dendrogram of relative diatom abundance from surface and bottom sediment slices. Data standardized by sample total, square root transformed, and using Bray–Curtis resemblance matrix. Bottoms slice community shown with “_b” and surface slice shown with “_s”. *HiddenNC* Hidden Lake in North Cascades, *HiddenMR* Hidden Lake in Mt. Rainier

The relative abundance of diatom communities identified from surface and bottom slices of the sediment cores (*n* = 10) were analyzed using a multivariate cluster analysis (Primer-E, v6) (Fig. 2). Surface and bottom relative abundance counts were all treated as independent samples. The cluster analysis indicates two insights. First, at eight of the ten lakes, the surface and bottom diatom communities resemble each other more so than a surface or bottom community from any other lake. The exceptions were Milk and Heather Lakes at Olympic National Park, where bottom and surface diatom communities resembled each other more so between these two lakes than they did between the surface and bottom sample within the same lake. Second, the most unique diatom communities of all lakes sampled were in Olympic National Park, and Hoh and Milk Lakes in particular (Fig. 2).

Results of the top and bottom diatom analysis identified only one lake with *A. formosa* (Hoh Lake) and *F. crotonensis* was not found at all. Milk Lake, also in OLYM, showed a high percent relative abundance of *Fragilaria tenera* in the top interval (38 %), a mesotrophic planktonic species which has been shown along with *A. formosa* to indicate enriched nutrient conditions in high elevation lakes in Colorado (Das et al. 2005). Similar effects have been recorded in experimental lake enrichments (Yang et al. 1996; Spitale et al. 2005). As a result, both Hoh and Milk lakes in OLYM were selected as two of the four lakes analyzed in more detail; the remaining two lakes included Copper Lake in NOCA and Snow Lake in MORA. At NOCA, Copper Lake was chosen because it had the highest summer bulk inorganic N deposition of any of the sites in this study. MORA’s Snow Lake, with the second highest summer bulk N deposition in the park, was chosen because although Eunice Lake had a higher summer bulk deposition value, diatom analyses of a recently collected core from Eunice Lake showed no evidence of either *A. formosa* or *F. crotonensis* (Drake 1998; Drake and Naiman 2000).

**Fig. 3:**
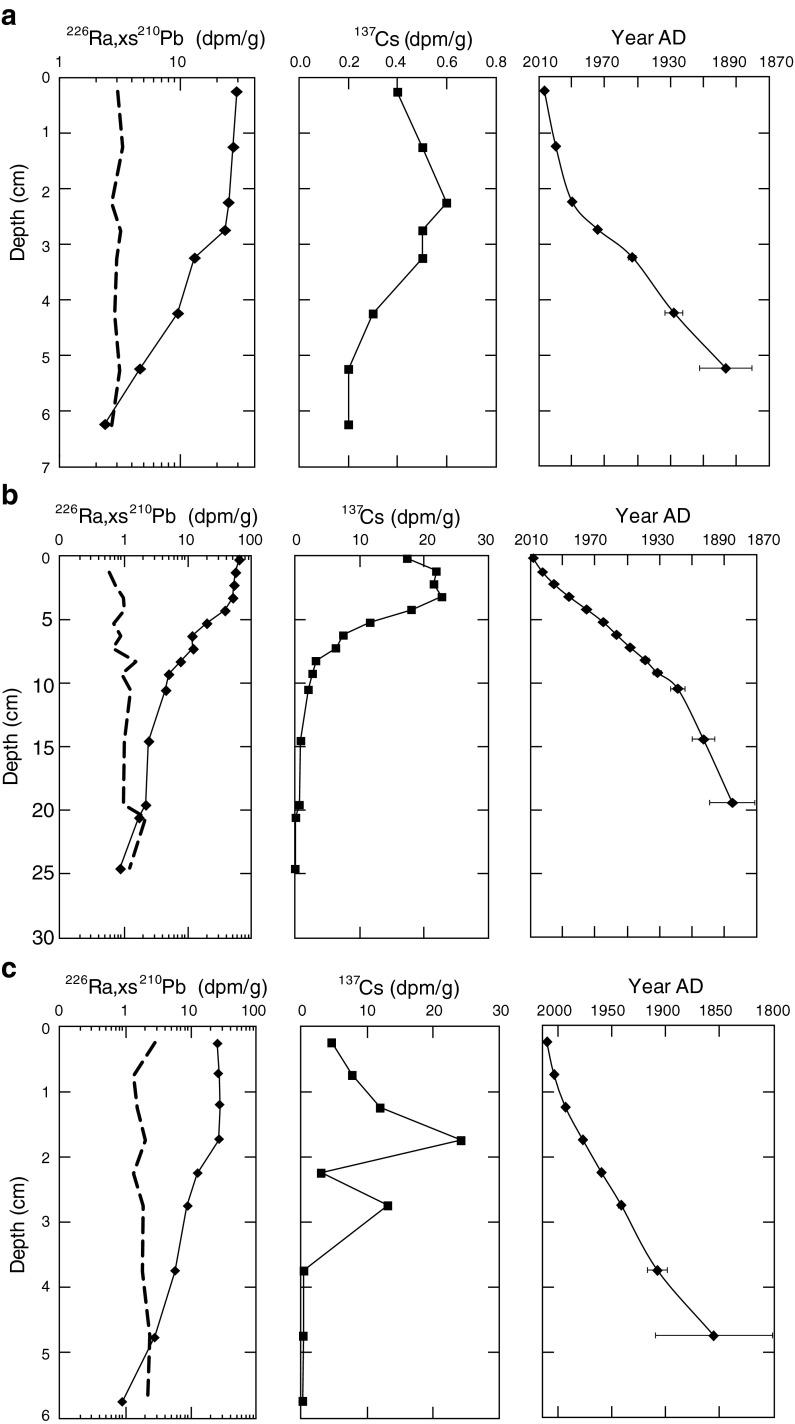

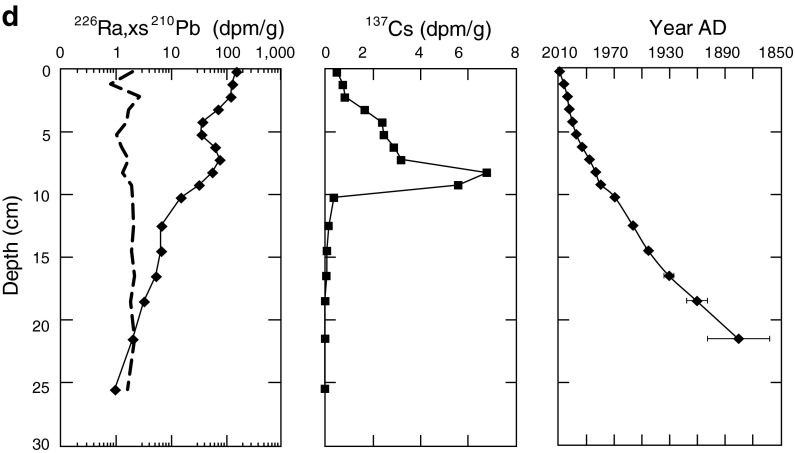
Depth profiles of ^226^Ra (*dashed*), xs^210^Pb (*solid*), ^137^Cs, and estimated age of sediment in **a** snow, **b** copper, **c** milk, and **d** Hoh Lakes. ^226^Ra line represents the parent supported 210Pb activity in each core. Age of sediment determined using the constant rate of supply model and data points represent average age ± 1 standard deviation

Sediment dates were derived using both ^137^Cs and excess ^210^Pb at these four sites (Fig. 3a–d). In general, we measured radionuclide activities to depths where profiles of ^226^Ra and total ^210^Pb converged indicating we reached background ^210^Pb levels in the four cores. The unsupported ^210^Pb (xs^210^Pb) was contained within the upper 10–20 cm of sediment at each of our four sites. Sedimentation rates varied across the four sites, with the 1900 time period occurring as shallow as 4–5 cm at Snow and Milk Lakes and as deep as ∼20 cm at Hoh Lake. Depths indicating the 1950s were approximately at 3.5, 4.5, 11, and 13 cm for Snow, Milk, Copper and Hoh Lakes, respectively. By combining xs^210^Pb data with ^137^Cs, which represent the height of atomic testing in 1963, confidence on the derived chronology was maximized where error bars (±1 SD) for dates in the upper slices were often less than 1 year, and increased in the deeper core sections (Fig. 3a–d). At Hoh Lake, a dip in the xs^210^Pb data indicate potential mixing of sediment of different ages as landslides have been largely absent in the basin. Despite this deviation in the data, we get a good fit to our data for determining the lake sedimentation rate (*r* ^2^ = 0.91) and our dates are comparable to the dates previously determined at this lake (Landers et al. 2008) (data not shown).

**Fig. 4:**
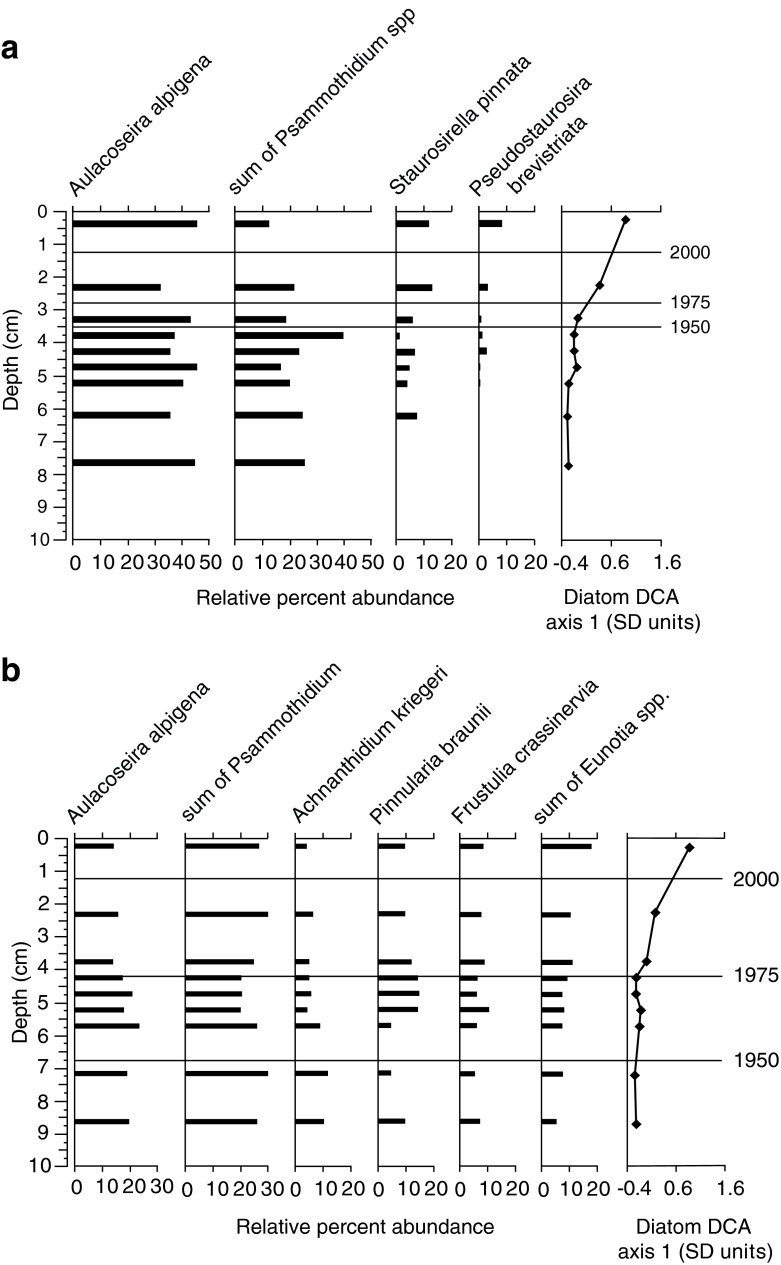

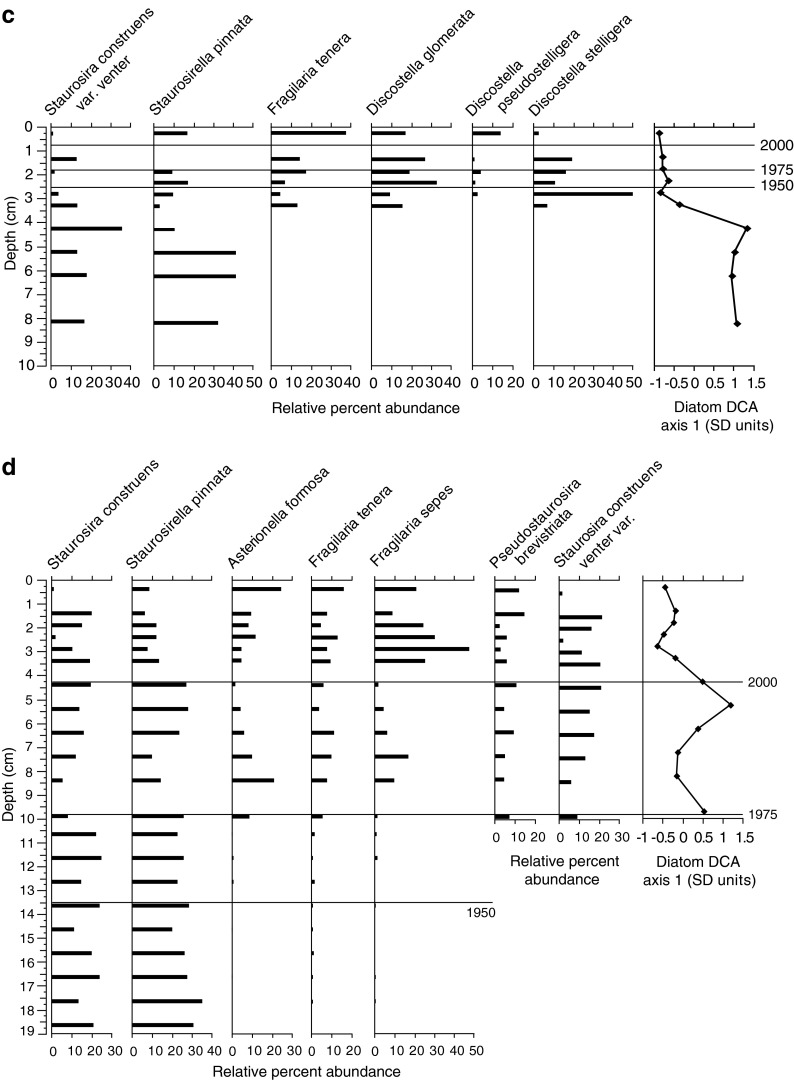
Depth profiles showing relative percent abundance of major diatom species and DCA axis 1 scores from **a** snow, **b** copper, **c** milk, and **d** Hoh Lakes. Note that for Hoh Lake, *P. brevistriata* and *S. construens* var*. venter* were only counted down to 10 cm

Diatom communities showed some interesting similarities and differences across the four focus sites (Fig. 4a–d). In general, diatom assemblages were very diverse with 120–140 different species identified within each lake core. Historical reconstructions were focused on the upper 10 cm of each core, except for Hoh Lake where samples down to 20 cm were analyzed (see details below). Diatom communities in Snow Lake at MORA were represented mainly by taxa common in undisturbed oligotrophic alpine lakes (Wolfe et al. 2001; Saros et al. 2011) and included *Aulacoseira alpigena*, *Psammothidium* spp., and small fragilarioids (dominated by *Pseudostaurosira brevistriata* and *Staurosirella pinnata*) (Fig. 4a). The *Psammothidium* spp. were abundant (13–40 % relative abundance), dominated by *Psammothidium curtissimum*, and very diverse, represented by 12 species, including some that have not been formerly described (Enache et al. 2013). Together, the above species identified for Snow Lake (Fig. 4) represented from 69 to 81 % of the total assemblages from each sediment interval in the upper 8 cm of the lake core. DCA axis 1 captures a first shift above 5 cm (∼the turn of last century) with a 25 % drop in *Psammothidium*, a clean cold water genus preferring sandy substrates, and a slight increase in small chain-forming fragilarioids suggesting a change to a more organic substrate. A second gradient shift is captured above 3 cm (∼1990) when the small fragilarioids (*Pseudostaurosia* and *Staurosirella* spp.) become ten times more abundant and *Psammothidium* spp. decline two to three times, suggesting further changes in substrate type and composition.

Copper Lake in NOCA showed diatom assemblages similar to Snow Lake with *Aulacoseira alpigena* being the most abundant species, followed by *Achnathaceae* species (dominated by *P. curtissimum* and *Achnanthidium kriegeri*) (Fig. 4b). Copper Lake also had a diverse and abundant *Psammothidium* spp population representing 14–29 % of the total relative abundance, and also includes some species not previously identified (Enache et al. 2013). In addition, Copper Lake showed a gradual increase in acid sensitive *Frustulia* and *Eunotia* spp. from the bottom of the core and a decline in *Aulacoseira alpigena* indicating a potential lowering of the lake pH (Camburn and Charles 2000) over the last century. These changes are also captured by the DCA axis 1 which summarizes an up to 2 times increase in *Eunotia* and *Frustulia* in the upper core intervals. The species shown in Fig. 4 for Copper Lake represent 74–86 % of total assemblages in the upper 9 cm of the core.

As indicated by the cluster analysis, diatom communities at Hoh and Milk Lakes in OLYM were very different than those at Copper and Snow Lakes (Fig. 4c–d). First, neither lake in OLYM had significant populations of *Aulacoseira*, *Achnanthidiums*, or *Psammothidium* spp. in any of the samples analyzed. At Milk Lake, assemblages were dominated by *Discostella* spp. (*Discostella glomerata*, *Discostella pseudostelligera*, and *Discostella stelligera*), which ranged from 22 to 62 % of the total assemblages in the upper 3.5 cm of sediment. Below this depth (down to 8.5 cm), *Discostella* spp. disappear (Fig. 4c). Changes in small fragilarioids, mainly *Staurosira construens* var*. venter* and *S. pinnata*, showed an opposite pattern, and this major change is captured by the DCA axis 1 (Fig. 4c). Below 3.5 cm, these species dominated (53–63 % of total assemblages) and decline as relative abundance of *Discostella* spp. increases above 3.5 cm depth. In addition, *F. tenera* increased along with increases in *Discostella* spp., and comprised 38 % of the assemblage in the uppermost sediment sample. This suggests changes in habitat availability with a shift toward more planktonic *Discostella* spp. potentially induced by limnological changes such as thermocline depth and light penetration (Hobbs et al. 2011), increased stratification and prolonged lake stability with less mixing (Enache et al. 2011; Rühland et al. 2008).

At Hoh Lake, the DCA axis one captures a major shift from assemblages with small fragilarioids to those with *A. formosa*, *F. tenera* and *Fragilaria sepes* dominants (Fig. 4d). Small fragilarioid species (dominated by *P. brevistriata, S. construens* var*. venter,* and *S. pinnata*) make up a large part of the total assemblages in the upper 20 cm of the core, at times comprising over 70 % of the total assemblage (Fig. 4d). In addition, *A. formosa*, *F. tenera*, and *F. sepes* appear above 10 cm (∼1970), and in general, increase towards the top of the core at the expense of small fragilarioids. Below 10 cm, the combined relative abundance of these three species is 2 % or less and they all disappear below 19–20 cm. In the upper 3 cm of the core, these three species alone comprise 40–60 % of the total assemblage. *F. tenera* and *F. sepes* are similar in morphology and ecology (Krammer and Lange-Bertalot 1991a) and increases in *F. tenera* are associated with increases in *A. formosa* from nutrient enrichment in some Colorado lakes (Das et al. 2005). Taken together, the increase in *A. formosa*, *F. tenera*, and *F. sepes* in the upper core at Hoh Lake is our clearest indication of nutrient enrichment across all our study lakes.

### Estimating a Critical Load for N

Based on recent increases in relative abundance of *A. formosa* and concurrent increases of *F. tenera* and *F. sepes* in Hoh Lake, we identify the 1969–1975 time period for estimating the level when N deposition reached a detrimental level. We see a similar increase in *F. tenera* in recent sediments at Milk Lake, located ∼53 km to the southeast of Hoh Lake; however, without the presence of other well documented indicators, we cannot conclusively say that Milk Lake diatoms are responding to increased deposition.

**Fig. 5:**
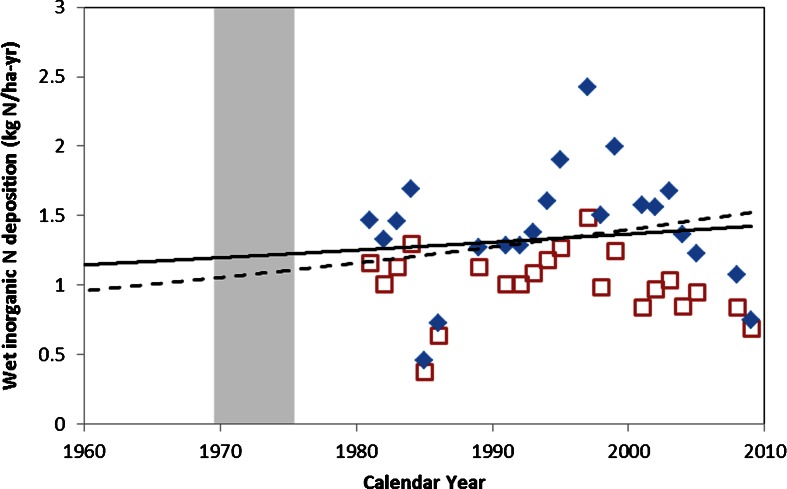
Annual wet inorganic N deposition from NADP station WA14 (Hoh Ranger station) (*open squares*) and elevation-corrected annual deposition (*solid diamonds*) using PRISM precipitation data at Hoh Lake and NADP annual N concentrations of inorganic N from station WA14. *Lines* represent the exponential fit to the elevation-corrected data with (*dashed line*) and without (*solid line*) a preindustrial anchor point of 0.5 kg N ha^−1^ year^−1^ (see text for more details). *Shaded* region represents the time period where diatom changes in the Hoh Lake sediment core were observed and a critical load was calculated

Elevation-corrected wet deposition of N at Hoh Lake was greater than wet deposition from the lower elevation at the closest NADP site, located ∼12 km southwest of Hoh Lake at Hoh Ranger Station (NADP station ID-WA14) during each year in the period of record (Fig. 5). The exponential fit of the elevation-corrected wet deposition data was solved for each year during the 1969–1975 of time period and averaged; we used two exponential fits of the data, one with and one without the anchor point used in previous studies (Baron 2006; Saros et al. 2011) to provide a range in the critical load. Using this procedure, we calculate a critical load of 1.0 ± 0.02 to 1.2 ± 0.01 kg N ha^−1^ year^−1^ for the exponential fit with and without the anchor point, respectively, for Hoh lake.

## Discussion

### Summer Bulk N Deposition

In general, summer bulk N deposition was highest in NOCA, lowest at OLYM, with MORA in between. The lower deposition at OLYM may be due to the fact that this park is located near the west coast of Washington and is mainly influenced by marine transport of N and sulfur. Edmonds et al. (1998) present a detailed description of the marine influence on precipitation chemistry in the Hoh River basin. In contrast, both MORA and NOCA are located downwind of urban and industrial areas, likely resulting in higher deposition at these parks. Fenn et al. (2003a) discuss that for western states, the likely primary source of atmospheric N is from urban and agricultural land uses. Furthermore, the proximity to local, primarily agricultural sources, appeared to be the best predictor of pesticides concentrations collected from these same, and similar, high elevation lakes in a study by Hageman et al. (2010) indicating that elevated gaseous N from fertilizer use is plausible in this area. We deployed the IER columns in summer only, when precipitation is low in Washington. Extending the measurement period to include the wetter months in spring and fall will allow for a better estimate of true annual rates. Given the extremely high level of snow fall in mountainous regions of Washington, the amount of annual high elevation deposition data in Washington is sparse at best. NADP does not have a high elevation deposition site in Washington and we are only aware of one site where year-round high elevation annual deposition is measured which is Paradise ranger station in Mt. Rainier National Park (Agren et al. 2013). The summer deposition measured using our IER columns (1.38 kg N ha^−1^ year^−1^) was higher than summer deposition reported by Agren et al. (2013) (0.62 kg N ha^−1^ year^−1^); however, the data are not directly comparable as we are measuring bulk deposition and the Paradise station only collects wet deposition.

### Lake Diatom Changes

Overall, only one (Hoh Lake) of our ten study lakes where cores were collected showed clear evidence of impacts from N deposition based on changes in sediment diatom communities. If we include *F. tenera* as another potential N deposition indicator, based upon previous findings (see above), we may potentially include Milk Lake within the N-impacted sites; however, the increase in *F. tenera* at Milk Lake takes place much earlier than at Hoh lake (∼1930s) and it is not clear why N deposition would induce changes in diatom communities decades earlier in this nearby lake. Furthermore, Milk Lake is unique among our study lakes as it is fed by glacial melt waters and, as its name implies, its surface water is dominated by glacial silt. In general, diatom changes in Milk Lake include declines in small fragilaroid taxa and subsequent increases in several planktonic species (*Discostella* spp. and *F. tenera*). Previous research has shown that glacial melt water can increase reactive N delivery to lakes (Saros et al. 2010), and that *D. stelligera* can be found under high N conditions (Arnett et al. 2012), as we see in Milk lake sediments around the 1930s. Increases in *D. stelligera* like those observed at Milk Lake, have been previously linked to the twentieth century climate warming in arctic and temperate pristine systems (Rühland et al. 2008; Enache et al. 2011). A similar increase in *D. stelligera* complex has been previously related to climate warming and associated prolonged ice-free season and water-column stratification at Emerald Lake, an alpine lake from WY, USA (Hobbs et al. 2010). Therefore, we conclude that changes at Milk Lake might be influenced by a combination of factors such as glacier activity potentially increasing N runoff, and changes in stratification leading to more stability, less mixing, and possibly higher temperatures. Additional research is needed to tease out the effects of these processes at Milk Lake before we can develop a critical load for this site.

Diatom data suggest that factors other than increases in nutrients are causing changes in diatoms at Snow and Copper lakes. At Snow Lake, relative abundances of several small fragilarioids double, while abundance of *Psammothidium* spp. sharply declines in the upper 2.5 cm of sediment (Fig. 4a). Although all these benthic species are successful when the ice free season is shorter and light attenuation low, with primary production concentrated in littoral zone (Smol et al. 2005; Smol and Douglas 2007), *Psammothidium* spp. have overall lower nutrient preferences than the small fragilarioid species (Krammer and Lange-Bertalot 1991a, b) and prefer more siltic substrates (Bukhtiyarova and Round 1996). Thus, declines in their abundance may imply changes to increased productivity and more organic matter deposition in the lake as ∼1980. At Copper Lake, the steady decrease in *A. alpigena* and increases in *Eunotia* and *Frustulia* spp. in the upper 8 cm of sediment may indicate effects from climate change with increased lake stratification and pH decline. In addition, there is evidence of changes from sandy to more organic substrate from 4 to 6 cm (∼1960s) where *Psammothidium* spp. temporally decrease and *Pinnularia* spp., who prefer more organic substrates than *Psammothidium* spp. (Krammer and Lange-Bertalot 1986) subsequently increase in relative abundance.

### Implications for Regional Critical Loads

At Hoh Lake, we conclude that diatom changes during the 1969–1975 correspond to changes in N delivery to the lake, and a critical load of 1.0–1.2 ± 0.01 kg N ha^−1^ year^−1^ is established for this site. This critical load is lower than the critical load of 1.4-1.5 kg N ha^−1^ year^−1^ reported in other western parks (Baron 2006; Saros et al. 2011), indicating that Hoh Lake might be more sensitive to increases in N deposition compared to regions further inland in the Western US. As a rough ‘test’ of our critical load estimate, we analyzed the variability of the PRISM-corrected wet deposition data from 1980 to 2009, with the assumption that because our diatom shifts took place well before this time period, every annual estimate from the PRISM-corrected data was already above the critical load. From this data, we calculated the 95 % lower confidence limit of the mean to be 1.2 kg N ha^−1^ year^−1^ which corresponds to the upper value in our critical load range.

The lack of increase of N enrichment indicators *A. formosa* and *F. crotonensis* at most of our sites would imply that one or more possibilities are occurring: (1) the critical load of N deposition has not yet been exceeded at the remainder of our study lakes, (2) that these lakes might not be N-limited, and (3) diatom communities indicative of N excess in the Pacific Northwest are different than the two species established from previous studies.

The calculated DIN/TP ratios in Table 3 were 1.5 or less (indicating N-limitation; Bergstrom 2010) at six of ten lakes sampled and four of ten lakes indicated possible co-limitation of N and P (Table 3). As none of the lakes indicated strict P limitation (DIN/TP, >3.4; Bergstrom 2010), we argue that all of our study lakes are potentially influenced by future increases in N deposition. However, it is hard to define N or P limitation based on the data from a single lake sample. Historical nutrient data from these ten lakes is sparse, and in many cases missing one of the parameters needed to calculate the DIN/TP ratio. Therefore, any conclusions made on N or P limitation should be taken in context of these data limitations.

There is the possibility that different or unknown diatom indicators exist in the Pacific Northwest compared to previous studies mainly in the Rockies. Since this paper presents some of the first data on this subject for the Pacific Northwest, more research is needed in order to fully answer this question.

The lack of response from nine of the ten lakes is an indication that these systems do not yet exceed their critical load, or we are not evaluating the correct indicators. Thus, monitoring of diatom communities into the future and examination of potential new diatom indicators for the Pacific Northwest should be focuses of future research. As we have downcore diatom data at snow, copper, and milk lakes, we attempt to determine a lower bound for the critical load at these sites by assuming that the critical loads are greater than the current amount of N deposition at these sites. Elevation-corrected deposition for these three lakes was calculated for 2000–2009 using PRISM precipitation and annual NADP concentration data following the same approach we used for Hoh Lake. Average elevation-corrected deposition for 2000–2009 for snow, copper, and milk lakes are 1.79, 2.02, and 0.62 kg N ha^−1^ year^−1^, respectively. Therefore we conclude that for these lakes, the critical loads are likely greater than these values.

### Processes Influencing Diatoms at Hoh Lake

It has been discussed in previous work using diatom-based critical loads that other potential factors besides increased deposition can alter nutrient dynamics and elicit responses from diatoms (Baron et al. 2000; Wolfe et al. 2003). Namely, watershed disturbance and introductions of non-native fish warrant discussion here. Watershed disturbance in the form of wildfires can increase nutrient delivery to aquatic ecosystems for many years after disturbance (Tiedemann et al. 1978; Betts and Jones 2009) through increased erosion potential of recently burned areas, as well as deposition and smoke and ash from nearby fires (Spencer et al. 2003). Olympic National Park has fairly complete fire history records since about 1916 to present (http://www.nps.gov/olym/parkmgmt/fire-history.htm; accessed 20th May 2013). The closest and largest fire to Hoh Lake was the Hoh Fire in 1978 that eventually grew up to ∼1,000 acres (http://www.nps.gov/olym/parkmgmt/fire-history.htm), but only about 80 acres was within the Hoh Lake Basin (William Baccus, personal communication). In addition, two large human-caused fires took place in the park in the 1980s and neither of these significant fire events were within the Hoh Lake basin. Therefore, watershed disturbance from fire is unlikely to have caused increases in nutrient delivery to the lake that would mimic the changes we observed in the diatom record of the lake.

Fish introductions can result in alterations to the natural nutrient cycling of lakes. For example, introduction of fish can reduce zooplankton populations, resulting in reduced grazing pressures (Carpenter et al. 1985; Wolfe et al. 2003). Fish can also release nutrients through excretion, resulting in increases in N and P into aquatic systems (Braband et al. 1990; Leavitt et al. 1994; Baron et al. 2000). Both the increase in nutrient inputs and reduction of zooplankton grazing can lead to increases in primary productivity of a lake. Hoh Lake has a large fish population consisting of Eastern Brook trout that were planted in 1957 (Hagen 1961). The increases in *A. formosa*, *F. tenera*, and *F. sepes* were not observed until 1969–1975, about 15 years after the lake was stocked. If fish introductions were impacting the ecology of Hoh Lake through alterations in N-cycling, changes would be expected to occur starting around the time of introduction (Leavitt et al. 1994; Baron et al. 2000). Furthermore, in a study at Mt. Rainier that looked at changes in diatom communities as a result of fish stocking showed that changes in the overall diatom community is common as a result of stocking, but the dominance of one to two species as a result of the introductions was not observed (Drake and Naiman 2000). In addition, *A. formosa*, *F. crotonensis*, *F. tenera*, and *F. sepes* were not present in either pre- or post- fish introduction in any of their diatom counts (Drake 1998). As there was an offset of more than a decade between stocking Hoh Lake and the changes, we saw in the sediment diatoms, coupled with the results from Mt. Rainier (Drake and Naiman 2000), changes in diatoms we observed could not be the result of fish introductions alone. Therefore, we conclude that the most probable reason for the diatom changes at Hoh Lake is from increased N deposition.

### N Deposition Sources to Hoh Lake

We suspect that atmospheric N deposition in Olympic National Park is possibly coming from N emissions in Asia and from marine shipping because the park is not downwind of any major urban or agricultural regions. Marine influence on precipitation chemistry in this area has been documented (Edmonds et al. 1998) so it is probable that N deposition would be related to marine transport and marine-based activities. In addition, sediment data collected from Hoh Lake during the Western Airborne Contaminants Assessment Project (WACAP) shows evidence of anthropogenic pollution from fossil fuel combustion. In this study, Hoh Lake sediment cores show evidence of large increases in spheroidal carbonaceous particle (SCP) flux beginning in 1920, peaking in 1940–1950, and then remains elevated since 1960 (Landers et al. 2008). SCPs are a byproduct of high temperature combustion of fossil fuels with a short atmospheric lifetime (2–7 days); therefore, increases in SCPs in the sediment record indicate exposure to fossil fuel combustion within a few hundred to a few thousand kilometers (Landers et al. 2008). Back-trajectories to identify atmospheric sources to Hoh Lake point to regional and transpacific sources of deposition to this lake.

Studies have looked at developing inventories of NO_x_ emissions for Asia (Kato and Akimoto 1992; Akimoto and Narita 1994; Ohara et al. 2007) and show that NO_*x*_ emissions in Asia have doubled during 1980–2000. Historic NO_*x*_ emissions for time periods prior to 1980 have shown that between 1940 and 1970, emissions in Asia, particularly eastern China, greatly increased as the economic opportunities began to grow within the region (van Aardenne et al. 2001). In addition, an analyses of tropospheric NO_*x*_ from 1960 to 2000 has shown that worldwide increases in NO_*x*_ were greatest during 1960–1980 time period and beginning around 1970–1975, the proportion of worldwide emissions from Asia has been increasing dramatically (van het Bolscher et al. 2008). It has been well established that emissions from eastern Asia can be transported long distances and influence air quality in the Western USA (Jaffe et al. 1999; Liang et al. 2004; Fischer et al. 2009, 2010). Therefore, it is plausible that NO_*x*_ emissions from eastern Asia may contribute N to Hoh Lake.

Olympic National Park lies close to the western coast of Washington, and Puget Sound is a major shipping hub for the Pacific Northwest. Since 1993, the Washington State Department of Ecology has been tracking the amount of vessel traffic into and out of Puget Sound (https://fortress.wa.gov/ecy/publications/SummaryPages/1308001.html). During this time period, the amount of cargo and passenger traffic has remained relatively steady, with tanker traffic rising. The total number of annual vessel entries into Washington waters is on the order of 6,000–7,000 during this time period. Currently, we are not aware of data prior to 1993 on the amount of vessel traffic in the Puget Sound region to establish trends prior to this time period. Worldwide NO_*x*_ emissions from global shipping can be large (∼7 Tg N/year; Corbett and Koehler 2003), and an inventory of global NO_*x*_ emissions from shipping has shown that between 1950 and 2000, NO_*x*_ has increased ∼4-fold, with the biggest increases from 1960 to 1980 (Eyring et al. 2005). We cannot say if these same emission trends apply for those vessels traveling into Washington waters, but these data suggest that we cannot rule out N emissions from marine shipping as a possible N source to Olympic National Park.

Overall, changes in terrestrial Asian and global shipping NO_*x*_ emissions have shown large increases during the time frame we observe changes in diatoms at Hoh Lake (1960–1980), and WACAP data shows that the lake is receiving increased anthropogenic deposition from local and marine sources. Taken together, it is plausible that these two sources as possible pathways of N deposition to the area; however, further research into emissions from Asia and marine shipping activities are needed in order to fully understand the possible sources of N deposition to Olympic National Park.

## Conclusions

Estimates of N deposition across 12 lakes in three National Parks were variable within and across Parks based on summer bulk deposition samples. In general, summer deposition estimates were greatest in North Cascades National Park and lowest in Olympic National Park. Diatom data from nine of ten lakes across three national parks in Washington State did not show clear impacts from N deposition based on indicator species previously reported. At Hoh Lake in Olympic National Park, shifts in diatom community compositions since 1969–1975 are indicative of N enrichment in the lake system. These community shifts allowed for the development of a critical load estimate for this lake ranging from 1.0 to 1.2 ± 0.01 kg N ha^−1^ year^−1^ for inorganic N. This study reconfirms that critical loads estimated from diatoms are lower than those estimated for other ecosystem endpoints (such as lichens, terrestrial plants, or lake acidification) (Pardo et al. 2011), suggesting, as Saros et al. (2011) have stated, that diatom-based critical loads can be used to provide broad ecosystem protection for lake ecosystems. Future work is needed to investigate the cause of N enrichment at Hoh Lake, although we suspect that NO_*x*_ from Asia and marine shipping to be important to this area. In addition, research into Pacific Northwest-specific diatom indicators is warranted. To date most work on developing diatom-based critical loads comes from the Rockies and Sierra Nevada mountains. We suspect the same species to be important here, as they are opportunists; however it is possible that other diatoms are sensitive to changes in N that we do not know about at this time.
